# Substance use disorders and risk of suicide in a general US population: a case control study

**DOI:** 10.1186/s13722-020-0181-1

**Published:** 2020-02-21

**Authors:** Frances L. Lynch, Edward L. Peterson, Christine Y. Lu, Yong Hu, Rebecca C. Rossom, Beth E. Waitzfelder, Ashli A. Owen-Smith, Samuel Hubley, Deepak Prabhakar, L. Keoki Williams, Arne Beck, Gregory E. Simon, Brian K. Ahmedani

**Affiliations:** 1 Kaiser Permanente Northwest, Center for Health Research, 3800 N Interstate Ave., Portland, OR 97227-1110 USA; 2 Public Health Sciences, Henry Ford Health System, Detroit, USA; 3 Department of Population Medicine, Harvard Medical School, Boston, USA; 4 Center for Health Policy and Health Services Research, Henry Ford Health System, 1 Ford Place, Suite 3A, Detroit, MI 48116 USA; 5 Behavioral Health Services, Henry Ford Health System, Detroit, USA; 6 HealthPartners, Institute for Education and Research, 8170 33rd Ave. South, PO Box 1524, MS 21111R, Bloomington, MN 55425 USA; 7 Kaiser Permanente Hawaii, Center for Health Research, 501 Alakawa Street, Suite 201, Honolulu, HI 96817 USA; 8 School of Public Health, Georgia State University, Urban Life Building, 140 Decatur St. Suite 434, Atlanta, GA 30303 USA; 9 Kaiser Permanente Georgia, Center for Clinical and Outcomes Research, Portland, USA; 10 Department of Family Medicine, University of Colorado, Denver, 3055 Roslyn Street, #100, Denver, CO 80238 USA; 11 Department of Internal Medicine, Henry Ford Health System, Detroit, USA; 12 Kaiser Permanente Colorado, Institute for Health Research, 2550 S. Parker Rd, Suite 200, Aurora, CO 80014 USA; 13 Kaiser Permanente Washington, Health Research Institute, 1730 Minor Ave, Suite 1600, Seattle, WA 98101-1466 USA; 14 Harvard Pilgrim Health Care Institute, Harvard Pilgrim Health System, Boston, USA

**Keywords:** Substance use disorders, Suicide, Alcohol use disorder, Drug use disorders

## Abstract

**Background:**

Prior research suggests that substance use disorders (SUDs) are associated with risk of suicide mortality, but most previous work has been conducted among Veterans Health Administration patients. Few studies have examined the relationship between SUDs and suicide mortality in general populations. Our study estimates the association of SUDs with suicide mortality in a general US population of men and women who receive care across eight integrated health systems.

**Methods:**

We conducted a case–control study using electronic health records and claims data from eight integrated health systems of the Mental Health Research Network. Participants were 2674 men and women who died by suicide between 2000–2013 and 267,400 matched controls. The main outcome was suicide mortality, assessed using data from the health systems and confirmed by state death data systems. Demographic and diagnostic data on substance use disorders and other health conditions were obtained from each health system. First, we compared descriptive statistics for cases and controls, including age, gender, income, and education. Next, we compared the rate of each substance use disorder category for cases and controls. Finally, we used conditional logistic regression models to estimate unadjusted and adjusted odds of suicide associated with each substance use disorder category.

**Results:**

All categories of substance use disorders were associated with increased risk of suicide mortality. Adjusted odds ratios ranged from 2.0 (CI 1.7, 2.3) for patients with tobacco use disorder only to 11.2 (CI 8.0, 15.6) for patients with multiple alcohol, drug, and tobacco use disorders. Substance use disorders were associated with increased relative risk of suicide for both women and men across all categories, but the relative risk was more pronounced in women.

**Conclusions:**

Substance use disorders are associated with significant risk of suicide mortality, especially for women, even after controlling for other important risk factors. Experiencing multiple substance use disorders is particularly risky. These findings suggest increased suicide risk screening and prevention efforts for individuals with substance use disorders are needed.

## Background

Suicide is a major public health concern in the United States; in 2014, 42,826 people died of suicide in the U.S., making it the country’s 10th leading cause of death [1]. Alcohol, tobacco, and drug use disorders have all been implicated in suicidal behavior in multiple studies [2–8], but most prior research has examined non-fatal suicidal behavior such as suicide ideation or suicide attempt [2, 4, 8].

Only a handful of studies have examined the relationship between substance use disorders (SUDs) and suicide mortality [2, 4, 5, 9, 10]. Of these, the majority have focused solely on the relationship between alcohol use disorder and suicide, with few studies exploring the potential role of other SUDs [2, 4]. Most of these studies do not consider whether multiple SUDs contribute greater risk than single SUDs [11]. In addition, most previous research has been limited to men, or to specific populations or risk groups such as veterans or psychiatric patients [12]. Most of these studies have also had relatively small samples, making it difficult to analyze results by subgroup, such as gender [2, 4, 8].

A few studies have examined the relationship between SUDs and suicide mortality in larger samples in the U.S [5, 9, 10]. The results of these studies suggest several important considerations. First, these analyses indicate that risk may differ by type of SUD, such as alcohol, tobacco, or other drugs [5, 9]. Second, they suggest that the strength of the association between SUDs and suicide may be greater for women than for men [9, 10]. Finally, these studies suggest that controlling for both physical health status and psychiatric comorbidity is important to understand the specific association between suicide mortality and SUDs [9]. However, these studies have all been conducted among Veterans Health Administration (VHA) patients, and thus their findings may not hold true for a broader general population. Specifically, members of the VHA population are likely to have different risk factors, such as combat related health issues, that may moderate the impact of SUDs.

Our study examined the relationship between SUDS and suicide mortality in a general population drawn from 8 large integrated health systems. Our goal was to estimate the relationship between alcohol, tobacco, and drug use disorders and the risk of suicide mortality. We estimated risk by single SUD diagnosis category (alcohol, tobacco, drug) and combinations of these categories. In addition, we examined stratified models by gender to examine whether the relationship between SUDs and suicide mortality was different for men and women. Our results may aid health systems in identifying people most at risk for suicide and in developing suicide prevention programs best suited to those at risk.

## Methods

### Design and data

We conducted a case–control study using data from 8 large health care systems participating in the Mental Health Research Network (MHRN), a research collaboration established in 2010 by the National Institute of Mental Health to improve understanding and management of mental health conditions through a closer connection between research, practice, and policy (http://hcsrn.org/mhrn/en/).

MHRN members participating in this study were HealthPartners (Minnesota), Harvard Pilgrim Health Care (Massachusetts), Henry Ford Health System (Michigan), and Kaiser Permanente health systems in Colorado, Georgia, Hawaii, Oregon, and Washington.

Cases included 2674 individuals who died by suicide between 2000 and 2013. Each health system maintains a research data warehouse organized according to the Health Care Systems Research Network Virtual Data Warehouse (VDW) model [13]. Data included in the VDW include insurance enrollment records, electronic health records (EHRs), insurance claims, pharmacy dispensings, state mortality records, and census-derived neighborhood characteristics. We determined suicide death initially through data from the VDW at each site using International Classification of Diseases, 9th revision (ICD-9) codes (X60-X84 and Y87) [14, 15], and confirmed this using state death certificate data. We obtained data on mortality from each state system using Social Security numbers or a combination of patient names, birthdates, and demographic information.

All included study subjects had been enrolled in one of the 8 health systems for at least 10 months in the year prior to suicide death. Each case was matched by time period (year of index date) and site to a randomly selected sample of 100 members who were also enrolled in the health system for at least 10 months in the same year as cases, for a total of 267,400 matched individuals from the general population of health system members. The date of suicide was used as the index date for cases and all matched controls.

We gathered Information on diagnoses, health care encounters, and other demographic information from the VDW at each site. These data included electronic health records (EHRs) from the health systems and insurance claims data for members of each health system [16–19]. All data were harmonized between sites as part of participation in the MHRN and are regularly assessed for quality and completeness. The Institutional Review Boards for each health system approved data use and research activities for this project.

### Measures

#### Suicide

The primary outcome was suicide death during the observation period. Individuals who died from suicide were cases. At each site, official regional mortality records were matched to all subjects by Social Security Numbers, patient names, birthdates, and demographic profiles. Deaths were identified and method of suicide was obtained using the regional mortality records and state death certificate data.

#### Substance use disorders

Substance use disorders (SUDs) were the primary predictors of interest. SUDs were identified using ICD-9 codes 303–305. We categorized SUD diagnoses into major type of SUD, focusing on the most common categories. Specifically, we created categories reflecting only one type of SUD diagnosis (alcohol only, drug only, tobacco only), and categories reflecting diagnoses of multiple types of SUD (alcohol + drug, alcohol + tobacco, drug + tobacco, alcohol + drug + tobacco). All SUD diagnoses were identified in VDW records of clinical encounters that occurred in the year prior to the index date [15].

#### Demographics

In adjusted analyses we included all available demographic indicators. For each study subject, we included age and gender from the VDW at each site and geocoded data on neighborhood income and education. Specifically, we created indicators of poverty and levels of education (college or higher vs. other). We were unable to include race or ethnicity for study subjects as these data were not available for all years of the study.

#### Other covariates

In adjusted analyses we also controlled for other factors that are known to be related to risk of suicide and that might confound the relationship between SUDs and risk of suicide. As psychiatric disorders are highly linked to suicide risk, we included an indicator of any psychiatric disorder. We extracted diagnoses for defined mental health diagnoses (ICD-9 codes 291–302, and 306–319). We also included the Charlson Comorbidity Index to control for non-psychiatric medical comorbidity such as cancer or cardiovascular disease [20]. The Charlson Index was calculated using ICD-9 codes from clinical diagnoses. All diagnoses were identified during clinical encounters that occurred in the year prior to the index date [21].

### Statistical analyses

First, we compared descriptive statistics for the cases and controls. We examined age, gender (male/female), income (proportion living in census blocks with ≥ 20% living below US poverty level), and education (proportion living in census blocks where ≥ 25% are college graduates). Next, we compared the rate of each SUD category for cases and controls. Finally, we used conditional logistic regression models to estimate unadjusted and adjusted odds of suicide associated with each SUD category. All models were conditional on site. We present two sets of analyses, unadjusted results and results adjusting for age, gender, poverty level, education, physical health status (Charlson Index), and psychiatric comorbidity. All analyses were conducted using SAS [22]. Statistical significance was assessed with a threshold of *p *= 0.05.

## Results

Table 1 compares cases and controls on demographic characteristics and SUD categories for the sample as a whole and separately by gender. Cases were significantly more likely than controls to be male (77.5% of cases were male vs. 47.5% of controls, *p *< 0.001) and to be older (average age of cases was 44.9 vs. 39.3 years for controls; *p *< 0.001). Cases were also more likely than controls to have psychiatric co-morbidities, as well as a higher Charlson Comorbidity Index score. We found no significant difference between cases and controls when we examined poverty level. We found a significantly higher level of education for female cases.

**Table 1:** Demographic and diagnostic categories

Category	All subjects [% or mean (SD)]			Males only [% or mean (SD)]			Females only [% or mean (SD)]		
	Controls	Cases	p-value	Controls	Cases	p-value	Controls	Cases	p-value
	N = 267,400	N = 2674		N = 126,980	N = 2072		N = 140,418	N = 602	
	% *[N]* or mean (SD)	% *[N]* or mean (SD)		% *[N]* or mean (SD)	*% [N]* or mean (SD)		% *[N]* or mean (SD)	% *[N]* or mean (SD)	
Male (ref. female)	*47.5[127,015]*	*77.5[2072]*	*0.008*	NA	NA	NA	NA	NA	NA
Low income	8.6 (8.6)	8.7 (8.6)	0.333	8.5 (8.5)	8.8 (8.6)	0.075	8.7 (8.6)	8.5 (8.4)	0.075
Higher education	*34.9* [93,323]	34.4 [920]	0.223	*34.9[44,316]*	*33.8[700]*	*0.007*	*34.8[48,865]*	*36.7[221]*	*0.007*
Age	*39.3 (21.9)*	*44.9 (19.0)*	*0.001*	*38.4* (21.8)	*50.4(19.3)*	*0.001*	*40.1(22.0)*	*48.2 (17.6)*	*0.001*
Psychiatric comorbidity	*12.6[33,692]*	*51.1[1366]*	*0.001*	*9.5[12,063]*	*46.3[959]*	*0.001*	*15.5[21,765]*	*67.6[407]*	*0.001*
Charlson index	*0.38 (0.99)*	*0.92 (1.70)*	*0.001*	*0.41(1.05)*	*0.98 (1.81)*	*0.001*	*0.36 (0.93)*	*0.71 (1.28)*	*0.001*
*Substance use disorder category*									
Alcohol only	*0.6[1604]*	*8.3[222]*	*0.001*	*0.8[1016]*	*8.5[176]*	*0.001*	*0.4[562]*	*8.0[48]*	*0.001*
Drug only	*0.4[1070]*	*3.8[102]*	*0.001*	*0.4[508]*	*2.5[52]*	*0.001*	*0.3[421]*	*5.1[31]*	*0.001*
Tobacco only	*5.5[14,707]*	*16.2[433]*	*0.001*	*5.4[6857]*	*15.9[329]*	*0.001*	*5.6[7863]*	*17.3[104]*	*0.001*
Alcohol + drug	*0.1[267]*	*2.9[78]*	*0.001*	*0.2[254]*	*2.8[58]*	*0.001*	*0.1[140]*	*3.4[20]*	*0.001*
Alcohol + tobacco	*0.3[802]*	*5.6[150]*	*0.001*	*0.4[508]*	*5.7[118]*	*0.001*	*0.2[281]*	*5.3[32]*	*0.001*
Drug + tobacco	*0.2[535]*	*2.3[62]*	*0.001*	*0.2[254]*	*1.8[37]*	*0.001*	*0.2[281]*	*4.2[25]*	*0.001*
Alcohol + drug + tobacco	*0.1[267]*	*3.6[96]*	*0.001*	*0.2[254]*	*3.3[68]*	*0.001*	*0.1[140]*	*4.6[28]*	*0.001*

Italic signifies *p* < 0.05

All individual SUD categories were significantly associated with suicide death. For instance, for the overall sample, the percent of cases diagnosed with alcohol use disorder only was more than 10 times greater than controls (8.3% of cases vs. 0.6% of controls, *p *< 0.001). Relative differences were greatest for those who used multiple categories of alcohol, tobacco and drugs (3.6% of cases vs. 0.1% of controls).

When we compared males and females separately we found that SUD was associated with suicide death for both genders. Male and female cases had a similar rate of some SUD diagnoses, for example 8.5% of male cases were diagnosed with an alcohol use disorder only (AUD) compared to 8% of female cases. However, the relative differences between cases and controls were different. For example, looking only at males, the percent of male cases diagnosed with AUD only was about 10 times greater than the percent diagnosed among male controls (8.5% of cases vs. 0.8% of controls, p < 0.001). In contrast, looking only at females, the percent of female cases diagnosed with AUD only was 20 times greater than the percent diagnosed among female controls (8.0% of cases vs. 0.4% of controls, *p *< 0.001). This pattern was consistent across most categories of SUD.

Table 2 reports results for conditional logistic regression for the entire sample. The first set of results presents unadjusted odds ratios; the second set presents results adjusted for age, gender, education, poverty level, physical health status, and psychiatric conditions. All SUD categories were significantly associated with suicide in both models. Unadjusted odds of suicide for different categories of SUDs ranged from 3.5 times increased risk for people with tobacco use disorder only (OR 3.5; CI 3.1, 4.0) to 30.7 times increased risk for people with alcohol, drug and tobacco use disorders (OR 30.7; CI 23.3, 40.6). Adjustment for demographics, psychiatric conditions and Charlson Index of physical health comorbidity reduced the odds ratios, but all categories of SUD continued to be associated with suicide at levels that were statistically significant. For example, odds of suicide adjusted for age, gender, education, poverty level, psychiatric conditions, and Charlson Index ranged from 2.0 times increased risk for people with tobacco use disorder only (OR 2.0; CI 1.7, 2.3) to 11.2 times for people with alcohol, drug and tobacco use disorders (OR 11.2; CI 8.0, 15.6). Conditional logistic regression results stratified by gender are presented in Table 3. Comparing male cases to male controls, adjusted odds of suicide for different categories of SUD ranged from 1.8 times increased risk for males with tobacco use disorder only (OR 1.8; CI 1.6,2.1) to 7.9 times for males with alcohol, drug and tobacco use disorders (OR 7.9; CI 5.6, 11.1). Comparing female cases to female controls, adjusted odds of suicide for different categories of SUD ranged from 2.5 times increased risk for females with tobacco use disorder only (OR 2.5; CI 1.9, 3.3) to 16.7 times for females with alcohol, drug and tobacco use disorders (OR 16.7; CI 7.9, 35.3). All categories of SUD continued to be significant after adjustments for other risk factors for both males and females.

**Table 2:** Risk of suicide by substance use disorder category, whole sample

Substance use disorder category	Odds ratio^a^			Adjusted odds (adjusted for age, gender, poverty level, education, Charlson index, psychiatric diagnoses)		
	OR	CI^b^	p-value	aOR	CI	p-value
Alcohol only	15.5	13.1, 18.4	0.001	5.8	4.7, 7.1	0.001
Drug only	11.3	8.8, 14.4	0.001	5.3	3.9, 7.0	0.001
Tobacco only	3.5	3.1, 4.0	0.001	2.0	1.7, 2.3	0.001
Alcohol + drug	21.8	16.2, 29.3	0.001	8.1	5.7, 11.5	0.001
Alcohol + tobacco	19.5	15.8, 24.2	0.001	6.1	4.8, 7.9	0.001
Drug + tobacco	14.1	10.2, 19.6	0.001	5.0	3.4, 7.4	0.001
Alcohol + drug + tobacco	30.7	23.3, 40.6	0.001	11.2	8.0, 15.6	0.001

^a^All conditional logistic regression models are conditional on site

^b^CI denotes 95% confidence interval

**Table 3:** Risk of suicide by substance use disorder category, by gender

Substance use disorder category	Odds ratio^a^			Adjusted odds (adjusted for age, education level, poverty level, Charlson index, psychiatric diagnoses)		
	OR	CI^b^	p-value	aOR	CI	p-value
Male only						
Alcohol only	11.7	9.6 14.3	0.001	4.6	3.7, 5.6	0.001
Drug only	8.2	6.1, 11.2	0.001	4.0	2.9, 5.4	0.001
Tobacco only	3.5	3.1, 4.1	0.001	1.8	1.6, 2.1	0.001
Alcohol + drug	14.5	10.1, 20.7	0.001	6.3	4.4, 9.0	0.001
Alcohol + tobacco	13.6	10.6, 17.5	0.001	5.5	4.2, 7.0	0.001
Drug + tobacco	10.4	6.8, 16.1	0.001	3.5	2.3, 5.4	0.001
Alcohol + drug + tobacco	19.5	13.9, 27.4	0.001	7.9	5.6, 11.1	0.001
Female only						
Alcohol only	22.4	14.8, 34.00	0.001	10.7	6.3, 18.1	0.001
Drug only	13.9	8.5, 22.8	0.001	5.2	2.9, 9.1	0.001
Tobacco only	3.8	3.0, 4.9	0.001	2.5	1.9, 3.3	0.001
Alcohol + drug	34.3	17.3, 68.0	0.001	11.8	5.4, 25.8	0.001
Alcohol + tobacco	23.0	13.8, 38.3	0.001	6.5	3.6. 11.8	0.001
Drug + tobacco	32.9	17.6, 61.4	0.001	10.4	4.9, 22.0	0.001
Alcohol + drug + tobacco	53.5	27.4, 104.3	0.001	16.7	7.9, 35.3	0.001

^a^All conditional logistic regression models are conditional on site

^b^CI denotes 95% confidence interval

## Discussion

We estimated the risk of suicide associated with SUDs for a general population sample of men and women who receive care in 8 large integrated health care systems spanning a variety of regions across the U.S. Our results suggest that SUDs are associated with significantly increased risk of suicide even after adjusting for other factors that are known to increase risk of suicide, such as psychiatric conditions or physical health comorbidity. We also examined the association of SUDs with risk of suicide for males and females separately. Our results indicate that all categories of SUD are associated with significantly increased risk of suicide for both males and females. Consistent with other studies and known epidemiology [1], we found that in general, men were more likely than women to have died from suicide. For men, the relative risk of suicide associated with SUDs was between 1.8 (tobacco only) and 7.9 (alcohol + drug + tobacco). For women, the relative risk of suicide associated with SUDs was between 2.5 (tobacco only) and 16.7 (alcohol + drug + tobacco). Finally, we found that having multiple SUDs was associated with significantly greater risk of suicide mortality than any of the other SUD categories.

The most comparable study to ours is a recent analysis using data from the VHA [9]. Bohnert and colleagues found increased risk associated with several categories of SUD amongst persons served at VHA facilities. Although the methods are not directly comparable to ours, due to differences in underlying study design, our results are generally in line with Bohnert’s findings that SUDs are consistently associated with an increased risk of suicide mortality. However, in the current study we also find that *even after controlling for other important risk factors* (e.g., psychiatric diagnoses), all categories of SUD are associated with increased risk of suicide. In contrast, Bohnert et al. find that after adjusting for other risks, increased risk of suicide death is only associated with some kinds of SUD. In addition, Bohnert et al. did not examine diagnosis of multiple types of SUD, while our work suggests that diagnosis of multiple SUDs is associated with increased risk of suicide beyond that associated with any one category of SUD.

Our results suggest that increased screening for suicide risk in persons identified with SUDs may be warranted. This might include screening for suicide risk at entry to substance use treatment programs, or ongoing monitoring for suicide risk during treatment. Although this type of screening or monitoring may be currently happening in some health care systems, more research is needed on systematic programs for monitoring and mitigating risk of suicide in persons with SUD. The small number of studies reported in the literature to date suggest that many addiction providers may not have formal training in suicide risk assessment, or may not consistently incorporate it into care [23]. In addition, health systems might want to consider suicide prevention screening for persons identified with SUDs in other settings such as primary care or emergency settings, where persons may be identified with SUD who are not currently in addiction treatment.

We find that all categories of SUD are associated with significant risk of suicide in both men and women even after controlling for known risk factors, such as psychiatric conditions, or physical health status. We also find that the relative risk associated with SUD is particularly high for women. This result is consistent with observations in the literature suggesting that women may be reluctant to seek care for substance use conditions compared to men, such that the women who *are* diagnosed have more severe conditions [24]. Our results suggest that health systems pay particular attention to risk of suicide in women with SUD. In addition, further research to explore potential differences in how SUD influences risk of suicide in men and women could help to shape future suicide screening and treatment efforts. Our results are consistent with, but somewhat different than, previous work. Similar to our findings, Bohnert et al. [9] found that after controlling for demographic factors and psychiatric comorbidity, the relative risk of SUD associated with suicide mortality was greater for women compared to men. However, we find a larger difference in relative risk of suicide for women compared with men associated with SUD. Bohnert et al. [9] included only patients served through VHA, and this limits the generalizability of results to a broader population. Our study includes a greater number of women who died of suicide than Bohnert et al. (602 in current study compared to 291 in the VHA study); thus, our study has more power to look at women separately from men.

Many persons with SUD have multiple diagnoses across different categories of SUD (e.g., alcohol, drugs) [25], yet few studies have examined the difference in risk of suicide mortality for single compared to multiple SUD diagnoses. We know of only one study from Mexico that has reported on this issue [26]. Consistent with Ocampo and colleagues, we found that diagnoses of multiple types of SUD are associated with greatly increased risk of suicide mortality for both men and women.

SUDs and some psychiatric conditions often occur together [27]. Yet, little previous research on risk of suicide mortality has been able to control for the effect of SUD in the context of other health conditions such as psychiatric conditions or physical health disorders [2]. The few studies that have addressed this issue suggest that specific psychiatric conditions, such as depression or bipolar disorder, may account for a significant portion of the relationship between SUD and suicide mortality [9, 28]. Our findings also suggest that psychiatric conditions likely play an important role in suicide mortality in those with SUD, but we also find that even after controlling for many types of psychiatric conditions, all categories of SUD remain important risk factors for suicide mortality.

Our results should be considered in light of several limitations. All persons included in our study were covered by private or public health insurance and were members of established integrated health systems. Therefore, the results may not apply to persons without insurance or those served by more fragmented systems. Although the sample of cases is relatively large for a study of suicide death, some of the subgroup analyses include small numbers of subjects, resulting in relatively wide confidence intervals. Because this is an observational study, we cannot rule out confounding due to unmeasured factors. In particular, we were not able to include some demographic variables that may be important moderators of the relationship between SUDs and suicide risk such as race or ethnicity, employment status, or marital status. We included adjustment for known psychiatric conditions, however, it is possible that some patients with SUD may have undiagnosed psychiatric conditions and that could account for some of the increased risk of SUD that we identified. We were not able to examine the risk associated with some specific individual types of drugs (e.g., marijuana), and it is possible that the risks may differ by type of drug. We also did not have measures of the severity of SUD. Those with more severe disorders may be driving the differences in suicide risk that we observe. Information on diagnoses of SUD are dependent on health care providers coding these diagnoses; thus some individuals with SUD may have been missed because health care providers did not recognize the disorder, or chose not to record the diagnosis. Thus, some controls may have undiagnosed SUD and this may make our results somewhat conservative. Although we included health system members from multiple states representing different geographic regions, not all U.S. states or healthcare settings were represented. In contrast to previous work, we did not match on age and gender. We limited matching to location and year, so that future analyses in this line of research could investigate variation in subgroups through analyses using interaction, stratification, and adjustment. In lieu of matching, this study adjusted the analyses for both age and gender. Although we used robust methods for identifying suicide death [29], it is possible that some deaths identified as suicide were accidental overdoses, as this can be difficult to distinguish in persons with some types of SUD [30].

Despite these limitations, our study provides one of the first reports of risk of suicide amongst individuals with SUDs in a general population. All SUD categories studied were associated with increased risk of suicide and our results suggest that health systems could increase screening and monitoring of suicide risk and plan services to help address suicide risk amongst persons with SUD. The focus of this study was examining the risk of suicide for persons with SUD. However, persons who are identified by health systems as at risk for suicide are also more likely at risk for SUD [31], therefore health systems may want to screen persons identified as at risk for suicide for SUD and to offer evidence-based treatment for SUD where warranted. Health systems may want to pay particular attention to how current services address suicide risk for women with SUD. Future research to better understand the significant relative risk of suicide amongst women with SUD could greatly aid health systems and providers to better serve women with SUD.

## Conclusions

Substance use disorders are associated with significant risk of suicide mortality, especially for women, even after controlling for other important risk factors. Experiencing multiple substance use disorders is particularly risky. These findings suggest the need for increased suicide risk screening and prevention efforts for individuals with substance use disorders.

## Data Availability

The datasets analyzed during the current study are available from the study PI, Brian Ahmedani, on reasonable request. Please contact Dr. Ahmedani at BAHMEDA1@hfhs.org.

## Abbreviations

EHR: Electronic health records
ICD: International Classification of Diseases
MHRN: Mental Health Research Network
SUD: Substance use disorders
AUD: Alcohol use disorder
VDW: Virtual Data Warehouse
VHA: Veterans Health Administration

## References

[1] CDC (Centers for Disease Control and Prevention). CDC suicide facts at a glance 2015; 2016. https://www.cdc.gov/violenceprevention/pdf/suicide-datasheet. Accessed 20 Aug 2017.

[2] Poorolajal J, Haghtalab T, Farhadi M, Darvishi N (2016). Substance use disorder and risk of suicidal ideation, suicide attempt and suicide death: a meta-analysis. Public Health.

[3] Poorolajal J, Darvishi N (2016). Smoking and suicide: a meta-analysis. PLoS ONE.

[4] Darvishi N, Farhadi M, Haghtalab T, Poorolajal J (2015). Alcohol-related risk of suicidal ideation, suicide attempt, and completed suicide: a meta-analysis. PLoS ONE.

[5] Bohnert KM, Ilgen MA, Mccarthy JF, Ignacio RV, Blow FC, Katz IR (2014). Tobacco use disorder and the risk of suicide mortality. Addiction.

[6] Borges G, Walters EE, Kessler RC (2000). Associations of substance use, abuse, and dependence with subsequent suicidal behavior. Am J Epidemiol.

[7] Conner KR, Ilgen MA, O’Connor RC, Platt S, Gordon J (2011). Substance use disorders and suicidal behaviour. The international handbook of suicide prevention: research, policy and practice.

[8] Wilcox HC, Conner KR, Caine ED (2004). Association of alcohol and drug use disorders and completed suicide: an empirical review of cohort studies. Drug Alcohol Depend.

[9] Bohnert KM, Ilgen MA, Louzon S, McCarthy JF, Katz IR (2017). Substance use disorders and the risk of suicide mortality among men and women in the US Veterans Health Administration. Addiction.

[10] Ilgen MA, Bohnert AS, Ignacio RV, McCarthy JF, Valenstein MM, Kim HM (2010). Psychiatric diagnoses and risk of suicide in veterans. Arch Gen Psychiatry.

[11] Ocampo R, Bojorquez I, Cortes M (2009). Substance use in suicides in Mexico: results of the epidemiological surveillance system of addictions, 1994–2006. Consumo de sustancias y suicidios en Mexico.

[12] Kim HM, Smith EG, Ganoczy D, Walters H, Stano CM, Ilgen MA (2012). Predictors of suicide in patient charts among patients with depression in the Veterans Health Administration health system: importance of prescription drug and alcohol abuse. J Clin Psychiatry.

[13] Ross TR, Ng D, Brown JS, Pardee R, Hornbrook MC (2014). The HMO research network virtual data warehouse: a public data model to support collaboration. eGEMs.

[14] World Health Organization (WHO). International classification of diseases, tenth revision; 2012. http://www.who.int/classifications/icd/en/.

[15] National Center for Health Statistics (2010). International classification of diseases, ninth revision, clinical modification.

[16] Ahmedani BK, Stewart C, Simon GE, Lynch F, Lu CY, Waitzfelder BE (2015). Racial/ethnic differences in health care visits made before suicide attempt across the United States. Med Care.

[17] Ahmedani BK, Simon GE, Stewart C, Beck A, Waitzfelder BE, Rossom R (2014). Health care contacts in the year before suicide death. J Gen Intern Med.

[18] Hornbrook MC, Hart G, Ellis JL, Bachman DJ, Ansell G, Greene SM (2005). Building a virtual cancer research organization. J Natl Cancer Inst Monogr.

[19] Go AS, Magid DJ, Wells B, Sung SH, Cassidy-Bushrow AE, Greenlee RT (2008). The cardiovascular research network: a new paradigm for cardiovascular quality and outcomes research. Circ Cardiovasc Qual Outcomes.

[20] Charlson ME, Pompei P, Ales KL, Mackenzie CR (1987). A new method of classifying prognostic comorbidity in longitudinal studies: development and validation. J Chronic Dis.

[21] Ahmedani BK, Peterson EL, Hu Y, Rossom RC, Lynch F, Lu CY (2017). Major physical health conditions and risk of suicide. Am J Prev Med.

[22] SAS Institute Inc. SAS/STAT users guide. 2008, version 9.2 ed. Cary: SAS Institute Inc.

[23] Ross J, Darke S, Kelly E, Hetherington K (2012). Suicide risk assessment practices: a national survey of generalist drug and alcohol residential rehabilitation services. Drug Alcohol Rev..

[24] Green CA (2006). Gender and use of substance abuse treatment services. Alcohol Res Health.

[25] Arnaout B, Petrakis IL (2008). Diagnosing co-morbid drug use in patients with alcohol use disorders. Alcohol Res Health.

[26] O’Campo R, Bojorquez I, Cortes M (2009). Substance use in suicides in Mexico: results of the epidemiological surveillance system of addictions, 1994–2006. Salud Publica Mex.

[27] CBHSQ (Center for Behavioral Health Statistics and Quality). Behavioral health trends in the United States: results from the 2014 National Survey on Drug Use and Health (HHS Publication No. SMA 15-4927, NSDUH Series H-50); 2015. http://www.samhsa.gov/data/.

[28] Simon GE, Hunkeler E, Fireman B, Lee JY, Savarino J (2007). Risk of suicide attempt and suicide death in patients treated for bipolar disorder. Bipolar Disord.

[29] Cowper DC, Kubal JD, Maynard C, Hynes DMA (2002). Primer and comparative review of major US mortality databases. Ann Epidemiol.

[30] Farrell M, Neeleman J, Griffiths P, Strang J (1996). Suicide and overdose among opiate addicts. Addiction.

[31] David-Ferdon C, Crosby AE, Caine ED, Hindman J, Reed J, Iskander J (2016). CDC grand rounds: preventing suicide through a comprehensive public health approach. Morb Mortal Wkly Rep.

